# Genistein in 1:1 Inclusion Complexes with Ramified Cyclodextrins: Theoretical, Physicochemical and Biological Evaluation

**DOI:** 10.3390/ijms15021962

**Published:** 2014-01-27

**Authors:** Corina Danciu, Codruta Soica, Mircea Oltean, Stefana Avram, Florin Borcan, Erzsebet Csanyi, Rita Ambrus, Istvan Zupko, Delia Muntean, Cristina A. Dehelean, Marius Craina, Ramona A. Popovici

**Affiliations:** 1 Faculty of Pharmacy, University of Medicine and Pharmacy “Victor Babeş”, Eftimie Murgu Square, No. 2, 300041 Timişoara, Romania; E-Mails: tiulea.corina@umft.ro (C.D.); avram.stefana@umft.ro (S.A.); fborcan@yahoo.com (F.B.); cadehelean@umft.ro (C.A.D.); 2 Faculty of Physics, Babes-Bolyai University, Kogalniceanu 1, 400084 Cluj-Napoca, Romania; E-Mail: mircea.oltean@gmail.com; 3 Department of Pharmaceutical Technology, University of Szeged, 6 Eotvos Str, H-6720 Szeged, Hungary; E-Mails: csanyi@pharm.u-szeged.hu (E.C.); arita@pharm.u-szeged.hu (R.A.); 4 Department of Pharmacodynamics and Biopharmacy, University of Szeged, 6 Eotvos Str, H-6720 Szeged, Hungary; E-Mail: zupko@pharm.u-szeged.hu; 5 Faculty of Medicine, University of Medicine and Pharmacy “Victor Babeş”, Eftimie Murgu Square, No. 2, 300041 Timişoara, Romania; E-Mails: deliacristimuntean@yahoo.com (D.M.); craina.marius@umft.ro (M.C.); 6 Faculty of Dental Medicine, University of Medicine and Pharmacy “Victor Babeş”, Eftimie Murgu Square, No. 2, 300041 Timişoara, Romania, E-Mail: ramonaamina@yahoo.com

**Keywords:** genistein, cyclodextrin, thermal analysis, X-ray diffraction, SEM, antibacterial, antiproliferative, antiangiogenic

## Abstract

Genistein is one of the most studied phytocompound in the class of isoflavones, presenting a notable estrogenic activity and *in vitro* and/or *in vivo* benefits in different types of cancer such as those of the bladder, kidney, lung, pancreatic, skin and endometrial cancer. A big inconvenience for drug development is low water solubility, which can be solved by using hydrophilic cyclodextrins. The aim of this study is to theoretically analyze, based on the interaction energy, the possibility of a complex formation between genistein (Gen) and three different ramified cyclodextrins (CD), using a 1:1 molar ratio Gen:CD. Theoretical data were correlated with a screening of both *in vitro* and *in vivo* activity. Proliferation of different human cancer cell lines, antimicrobial activity and angiogenesis behavior was analyzed in order to see if complexation has a beneficial effect for any of the above mentioned activities and if so, which of the three CDs is the most suitable for the incorporation of genistein, and which may lead to future improved pharmaceutical formulations. Results showed antiproliferative activity with different *IC*_50_ values for all tested cell lines, remarkable antimicrobial activity on *Bacillus subtilis* and antiangiogenic activity as revealed by CAM assay. Differences regarding the intensity of the activity for pure and the three Gen complexes were noticed as explained in the text. The data represent a proof that the three CDs can be used for furtherer research towards practical use in the pharmaceutical and medical field.

## 1. Introduction

It is well known that natural compounds, the base of folk medicine and, consequently, pharmacognosy, represent excellent sources of inspiration for modern medicine, continuously offering a wellspring of new remedies [1]. Genistein (Gen) is one of the most studied phytocompounds in the class of isoflavones [2]. The interest in the Gen’s mechanism of action was directly associated with the discovery of its strong estrogenic activity [3,4]. Gen is best represented in soybean, the vegetal product from *Glycine max* (L.) Merr*. Fabaceae* Family, but there are also other medicinal plants like red clover, lucerne or dyer’s broom that contain important amounts of Gen [5,6]. The concentration of this isoflavone in most types of soy food was found to be between 1–2 mg/g [7]. It has been noticed that oriental populations, who have low rates of breast and prostate cancer, consume 20–80 mg of Gen daily, almost entirely derived from soy, whereas the dietary intake of Gen in the US or Western Europe is only 1–3 mg daily [8,9]. *In vitro* and/or *in vivo* benefits of the compound were also reported in other types of cancer such as those of the bladder, kidney, lung, pancreatic, skin, and endometrial cancer [2]. Additionally, dietary supplements containing Gen are intensively used as means to counteract symptoms of menopause [10,11]. Other health benefits consist of a cardioprotective effect, improved arterial elasticity, antioxidant capacity, anti-inflammatory and anti-allergic potential [5,12–15]. Furthermore, recently Gen has been proposed as therapy for Sanfilippo disease [16]. In combination with an anti-CD19, it was also found to be an active agent for the control of leukemic human B lymphocytes [17].

Contrary to all these pluses, the chemistry of this compound exhibits a big minus: low water solubility, presumably related to its low bioavailability. Therefore, much interest has been focused on the design of analogs and/or conjugates with optimized physicochemical properties [18]. One of the many approaches involves the incorporation in cyclodextrins (CD). CDs are cyclooligosaccharides with the ability to form host-guest inclusion complexes with a wide variety of molecules [19]. One of the most important benefits resides in increasing the water solubility of lipophilic agents [20]. According to this property, they have several applications in the pharmaceutical field, especially for preparation of low soluble biologically active molecules [21]. Genistein, the compound involved in the formation of the complex, satisfies all the necessary conditions for its formation [22]. The successful incorporation of Gen in native cyclodextrins: β- and γ-cyclodextrin was previously reported, while α-CD did not form a stable complex [23]. Furthermore, using animal models, enhanced bioavailability and better anti-inflammatory properties were detected for Gen:CD inclusion complexes [5,24].

In our previous work, our main goal was to improve the most the water solubility for genistein, in order to be able to perform biological tests; therefore, a 1:2 molar ratio was used. In the current paper, we accomplished phase solubility studies wich revealed a 1:1 complexation ratio. The use of a smaller amount of cyclodextrin seems beneficial for future drug formulations, because it reduces the total amount of solid substance necessary for administration and the toxicity potential of the product.

The aim of this study is to theoretically analyze, based on the interaction energies, the possibility of complex formation between Gen and three different ramified CDs, using a 1:1 molar ratio Gen:CD. Theoretical data were further evaluated against experimental results following a screening of both *in vitro* and *in vivo* activity: proliferation on different human cancer cell lines, antimicrobial activity and antiangiogenic behavior. Our ultimate goal was the detection of a possible beneficial effect of cyclodextrin complexation as well as the selection of the most suitable cyclodextrin for Gen encapsulation and optimized pharmaceutical formulations.

## 2. Results and Discussion

**Figure 1.**
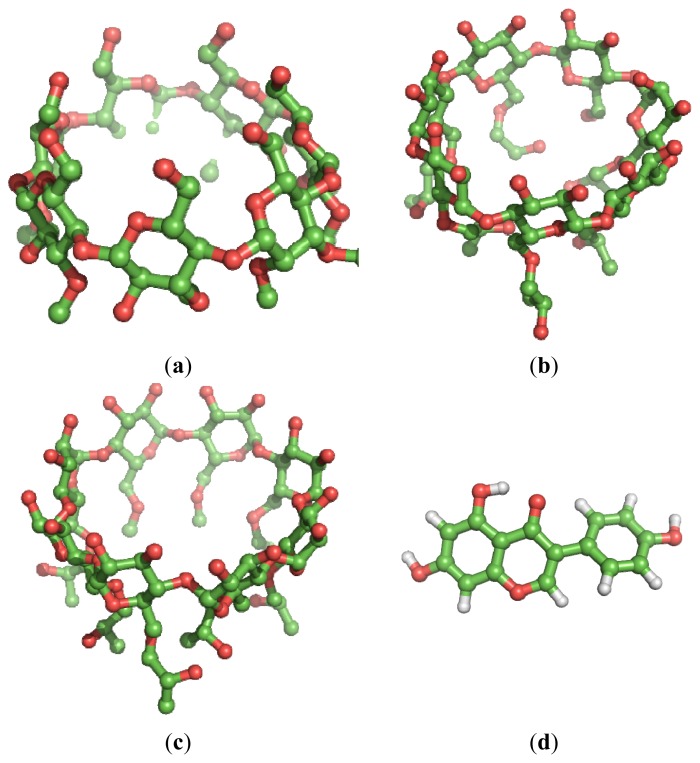
3D representation of CDs clusters and Gen as follows: (**a**) RAMEB; (**b**) HPBCD; (**c**) HPGCD and (**d**) Genistein. Hydrogens are omitted for clarity.

Quantum chemical calculations are used in this paper in order to theoretically investigate the possibility of complex formation between Gen and the three CDs: randomly methylated β-cyclodextrin (RAMEB), hydroxypropyl β-cyclodextrin (HPBCD) and hydroxypropyl γ-cyclodextrin (HPGCD) (Figure 1) analyzing the behavior in gas phase, in the solvent used for the solubilisation of active agents for all the mentioned assays, namely dimethyl sulfoxide (DMSO) and in water.

Comparing the binding energies, one can notice that the values are in direct relationship with the size of the complex (*i.e.*, these energies increase with the size of the complexes). Their stability order is as follows: Gen-HPGCD, Gen-HPBCD and Gen-RAMEB. Comparing the differences between the non-corrected energies and the corrected ones, using counterpoise scheme (CP) proposed by Boys and Bernardi [25], we can state that these binding energies are much more consistent if we take into account the effect of the basis sets superposition error (BSSE) (Table 1). The energetic values of the interaction energies place themselves within the normal range of values, applicable to the molecules, which exhibit van der Waals interactions combined with the H–bonding interactions.

The theory upholds that the higher the interaction energy, the more stable the complex is. This property can lead to a more elaborate dissociation mechanism for Gen from the cavity of the three CDs taken into study in order to be able to interact with the tested systems. The results presented in Table 1, in gas phase, DMSO solution and water suggest that, based on the previously issued idea, Gen-RAMEB would be the most proper complex, in terms of both increased water solubility of Gen and lower interaction energy. As we can see from the values in Table 1, the correlations between the binding energy calculated in gas phase, DMSO solution and water suggest that the reaction mechanism of dissociation is no very much influenced by the solvent (DMSO or water). The differences between the interaction energies computed in gas phase and solvent (DMSO or water) vary between 5–6 kcal/mol for uncorrected energies, and 1–2 kcal/mol for corrected ones. However, the fact that the binding energies calculated in liquid solvent (DMSO or water) are lower than the counterparts calculated in the gas phase is a clear indication of the competition between the H-bonding of Gen with the cyclodextrin cavity and the solvent. Moreover, the range of values for the differences on corrected results is close to the limit of 1 kcal/mol, important from the point of view of the accuracy of the chemical computations, at this level of theory (see computational details).

In addition, it is important to highlight that the purpose of this theoretical analysis was to determine the capacity of complex formation, and to analyze the relative stability between Gen and the three CDs. These relations resulted from their stability are directly linked to their property (characteristic) to act as drug-delivery systems.

Phase solubility studies of Gen in aqueous solutions of cyclodextrin at room temperature revealed an A_L_-type phase solubility diagram. According to literature [26], a slope < 1 indicates the formation of a 1:1 complex. The apparent stability constants were calculated for the complexes of Gen with all three cyclodextrins (Table 2), the values revealing a relatively strong interaction between Gen and cyclodextrins.

In order to correlate theory with experimental data, we have highlighted the changes in the physicochemical properties of pure Gen *versus* Gen:CDs complexes in 1:1 molar ratio, proving the occurrence of real complexation.

A number of consecrated techniques (X-ray diffraction, SEM analysis and DSC) are used in order to show alterations of the physico-chemical properties of the complex as compared to genistein or CD, respectively; these techniques are already mentioned by different sources [27–29] as reliable methods in the field of cyclodextrin complexation. Although neither of these methods can prove the actual Gen inclusion inside the cavity of the cyclodextrin, they offer an undeniable proof of the interaction that takes place between Gen and the specific cyclodextrin (HPGCD, HPBCD, RAMEB). This interaction could be the result of an inclusion complexation between the two components or an external association of the two molecules, Gen and CD; regardless of the nature of this interaction, the Gen final physico-chemical properties as well as its pharmacological activity are markedly improved due to this supramolecular assembly.

Differential scanning calorimetry (DSC) is a thermal analysis which provides information in terms of thermal stability or phase transition for CDs complexes, and, associated with other procedures, it can be a valuable tool in revealing a true complex formation as well as the molar ratio CD:active principle.

**Figure 2.**
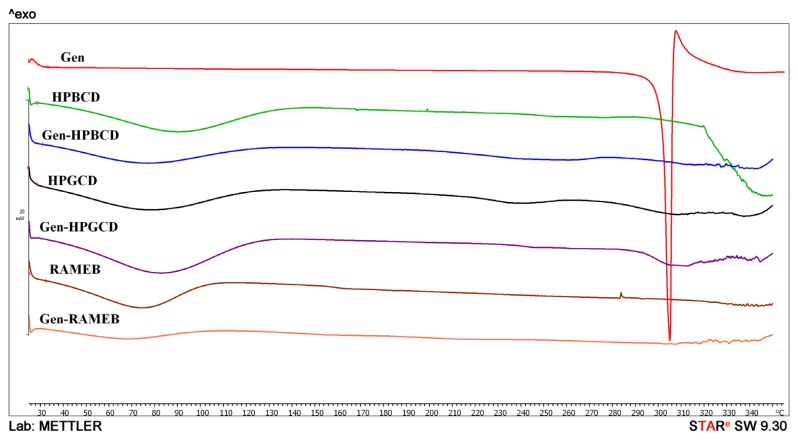
Differential scanning calorimetry (DSC) for Gen; and its’s 1:1 CDs complexes, respectively pure CDs.

Figure 2a,b shows the DSC curves for Gen and its complexes with the three CDs. Gen reveals a single sharp endothermic peak around 303 °C, corresponding to its melting; the DSC curves of all three CDs alone are depicted for comparison to Gen complexes. All three CDs reveal a water loss process before 100 °C, profile preserved by the CD:Gen complexes. Gen endothermic peak disappears completely for all complexes. The decomposition of the complexes starts around 320 °C.

Furthermore 1:2 Gen: CDs inclusion complexes were subjected to this type of analysis [5]. The sharp endothermic peak of Gen was attributed to its melting point, which corroborates with the literature specifications for Gen. For cyclodextrins, DSC curves show only a dehydration process before 100 °C and a decomposition process starting from 320 °C. The DSC plots of the 1:1 complexes reveal the same dehydration process but the endothermic peak of Gen disappears completely, which indicates that Gen was trapped inside the cyclodextrin’s cavity or that an amorphous solid product was formed; also both processes could be involved.

X-ray diffraction analysis is often involved in the assessment of a true inclusion complex formation, the analysis comparing the positions and intensity of the peaks of the pure compound with the ones attributed to the prepared complexes.

**Figure 3.**
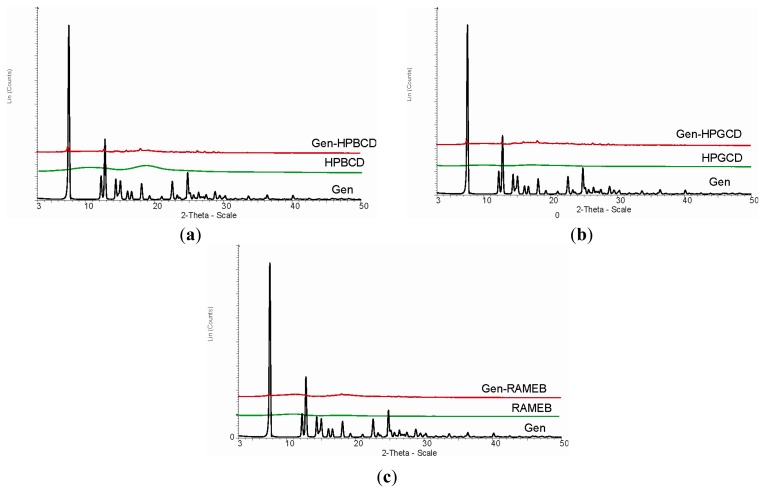
X-ray diffractograms for Gen and its 1:1 complexes as follows: (**a**) Gen-HPBCD; (**b**) Gen-HPGCD; (**c**) Gen-RAMEB.

Figure 3a–c exhibits the X-ray diffractograms for Gen and its 1:1 complexes with the three CDs involved in the study. Gen shows sharp, intense peaks, due to its crystalline nature; all three CDs present a flat diffractogram, while the 1:1 complexes present a massive reduction of the peaks’ intensity and also a shift of their positions.

The complexes reveal an almost flat X-ray diffractogram, with a significant decrease in the Gen peaks’ intensity, practically proving themselves as amorphous substances; only a very small fraction of crystalline Gen can be seen in the complexes difractograms, the majority of the peaks having disappeared completely. This type of curve can be interpreted as a proof of a real inclusion of Gen inside the cyclodextrin’s cavity, which hinders the crystalline nature of the active principle.

Scanning electron microscopy (SEM) is frequently involved in the characterization of cyclodextrin inclusion complexes; in spite of the fact that the method cannot provide a 100% confirmation of a complex formation, it contributes to the identification of the presence of a single component in the analyzed product.

**Figure 4.**
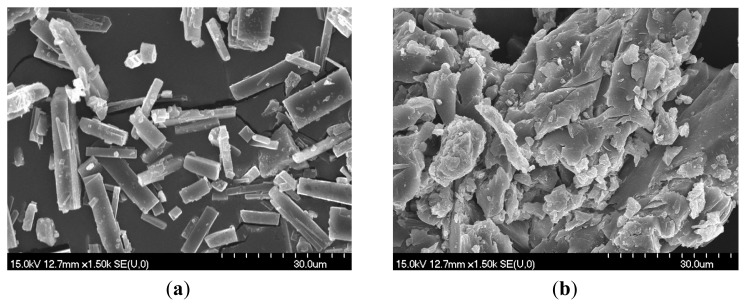

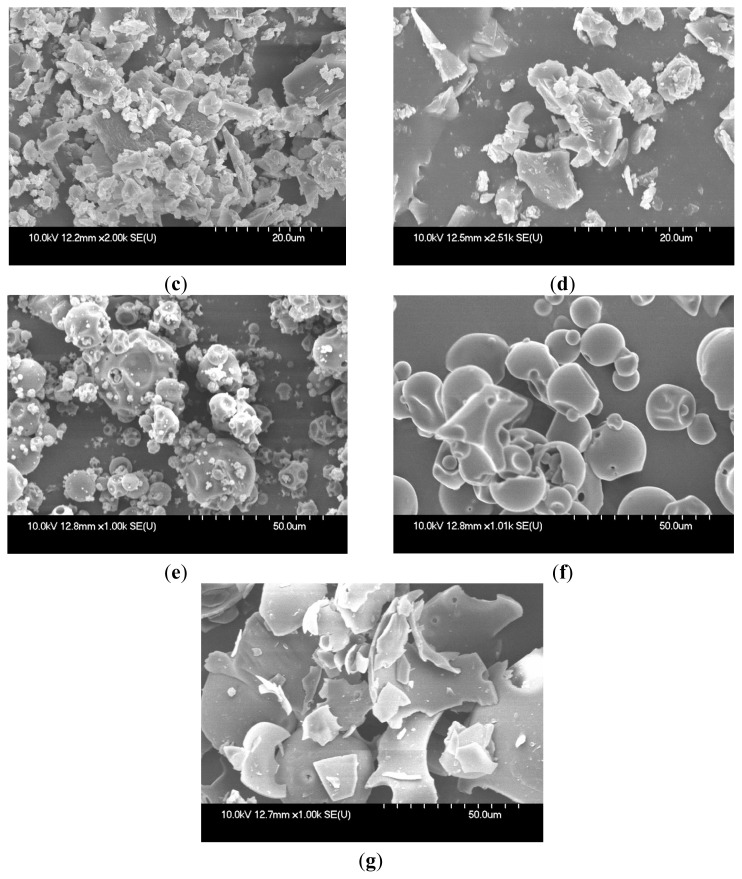
Scanning electron microscopy (SEM) assay for: (**a**) Gen; (**b**) Gen: HPBCD; (**c**) Gen: HPGCD; (**d**) Gen: RAMEB; (**e**) HPBCD; (**f**) HPGCD; (**g**) RAMEB.

Figure 4a–d depicts Scanning electron microscopy (SEM) images of Gen and its 1:1 complexes; while Gen reveals distinct crystals of a specific shape, its CDs complexes are generally formed by amorphous, conglomerated particles.

Gen, a crystalline substance, is formed by particles with distinct shape and size; its 1:1 complexes show a significant change of morphology, presenting irregular particles, usually aggregated, which indicates an amorphous product resulted following complexation.

**Figure 5.**
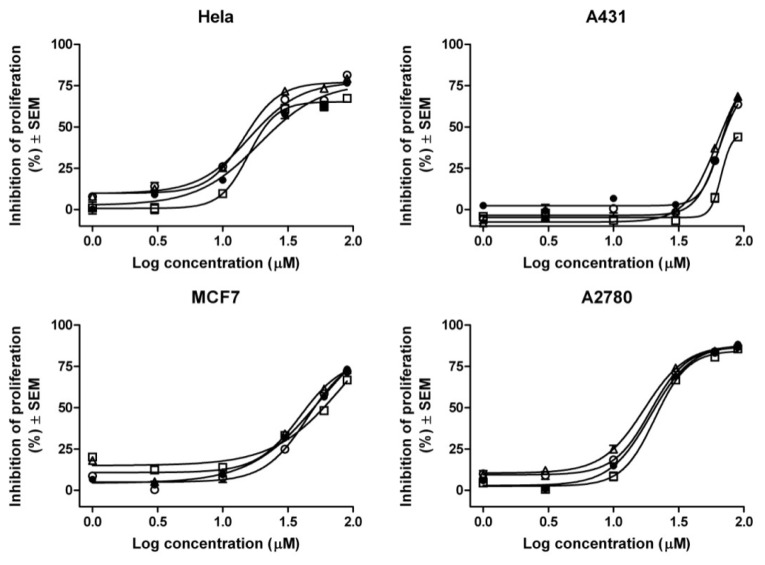
Dose-response curves of genistein (●) and the tested complexes prepared with RAMEB (□), HPBCD (Δ) and HPGCD (○) (1, 3, 10, 30, 60 and 90 μM) on four different human cancer cell lines: Hela, A431, MCF-7, A2780.

One of our aims was to screen the antiproliferative and/or cytotoxic effect of Gen and its cyclodextrin complexes on four human cancer cell lines: MCF-7, HeLa, A2780 and A431. Results show that pure Gen is mostly active on human ovarian carcinoma cell line A2780 followed by HeLa while all the other cell lines proved less sensitive. Incorporation inside cyclodextrins brings changes in the antiproliferative action, with respect to the cell line: all three complexes present a slightly increased antiproliferative activity for HeLa cell line, compared to pure Gen. The *IC*_50_ values are very similar to the ones corresponding to A2780 cell line, where complexation also seems to be a favorable option; an exception is represented by Gen:RAMEB which shows a slightly increased *IC*_50_ value. In this case, higher *IC*_50_ values, thus lower sensitivity, can be noticed in case of MCF-7 and A431 cell lines. For these two cell lines, MCF-7 and A431, Gen:HPBCD had the strongest antiproliferative activity. Results can be seen in Figure 5. *IC*_50_ values are presented in Table 3. CDs alone did not present any significant antiproliferative activity.

In terms of the *in vitro* antiproliferative effect, some correlations to our reported results are presented as follows: Gen is known for its dual mechanism on MCF-7 breast cancer cell line. The isoflavone presents proliferative effect at nanomolar concentrations and antiproliferative mechanism at micromolar concentrations. *IC*_50_ for this cell line, after 24 h of incubation was reported between 28–55 μM, depending on the experimental conditions [30,31]. Additionally, it was noticed that Gen increases the sensitivity of cisplatin in case of A2780 cell line [32]. The natural compound was also reported as a pro-apoptotic agent on this cell line [33]. For HeLa cervical adenocarcinoma, successful results were found when Gen was combined with ionizing radiation or with cisplatin but the phytocompound alone also presented an antiproliferative effect [34,35]. In the case of A431, skin epidermoid carcinoma, Gen 150 μM was reported to significantly decrease the phosphotyrosine level of EGF receptor [3].

The CDs alone had none or decreased antiproliferative effect. This observation is also supported by the group of Hipler *et al.* [36] who reported that in a series of experiments CDs alone in concentrations up to 0.1% (*w*/*v*) did not show any effect on HaCaT keratinocytes [36]. Some papers reported that CDs improve drug penetration into the cells by increasing cellular uptake, a strongly correlated phenomenon to the enhancement of cytotoxic activity of inclusion complexes [37–39]. During the present study, we did not detect any significant improvement after cyclodextrin incorporation. This situation was previously reported in the literature concluding that the rate of CD inclusion complexes uptake depends on the type of cancerous cell, membrane properties and metabolic state of the cell [40–45].

Gen and its CDs complexes were further tested for antimicrobial activity (Table 4). Results show that among the selected bacterial strains, the Gram positive *B. subtilis* is the only sensitive bacteria. CDs complexes manifested an increased activity, determining a 16 mm inhibition zone for Gen:RAMEB and Gen:HPBCD, respectively, and a 17 mm inhibition zone for Gen:HPGCD complex compared to pure Gen, case in which a 15 mm inhibition zone was detected. In terms of bacterial sensitivity, we can state that incorporation of Gen in ramified CDs is an efficient method to improve its activity. Slightly sensitive bacteria for Gen and its complexes were also *S. aureus* and *E. faecalis.* In the MIC (minimum inhibitory concentration) test, inhibition of analyzed bacteria using 0.63, 1.25, 2.5, 5 and 10 mM Gen and its CD complexes, respectively, was observed only for *B. subtilis* at the concentration of 10 mM, both for pure Gen and its complexes. CDs alone did not present any antimicrobial activity.

Among the tested bacterial strains, Gen and its CDs complexes, at the concentration of 10 mM, presented a significant antibacterial activity for *Bacillus subtilis*. Our findings corroborate with the results reported by Ulanowska *et al.* [44], who showed that the growth rate of *Bacillus subtilis* was approximately two times decreased when incubated with Gen 100 μM [44]. Another study showed that 100 μM Gen, at lower incubation time (*i.e.*, 8–10 h) is an active substance on some Gram positive bacteria (*i.e*., *Staphylococcus aureus*, *Bacillus anthracis*) when using a low concentration of bacteria (*i.e*., 1 × 10^3^). The observation is not valid for Gram negative bacteria [46]. On the other hand, total isoflavone extract from *Cicer arietinum* showed higher inhibitory activities for Gram negative strains compared to Gram positive strains [47]. *Escherichia coli* and *Shigella sonnei* were previously reported as resistant to Gen when using lower concentrations (*i.e.*, Gen 100 μM) [46,48]. *Pseudomonas aeruginosa* was resistant to Gen and its complexes. The group of Ulanowska *et al.* [44] reported that this bacterial strain is sensitive to another structure very similar to the one of Gen, namely the isoflavone daidzein [44]. Gen complexes with the three CDs showed a slight improvement of the antibacterial activity on the sensitive strain *B. subtilis*. Among the three complexes, Gen:HPGCD was the most active. Increased antibacterial activity after incorporation in CDs was reported for some well-known agents like trimethoprim, chlorhexidine, miconazole [49–51]. The CDs alone had no significant antibacterial activity.

Finally, for the same samples (*i.e.*, Gen and the complexes obtained with the three CDs), *in vivo* screening was performed using the chorioallantoic membrane of the chicken embryo. The samples were applied on the seventh day of incubation, in order to evaluate their impact on the rapid growing vascular plexus of the membrane. The evaluation was performed *in ovo* and *ex ovo*, by stereomicroscopy, observing the modifications induced at the level of blood vessels around the area of application.

**Figure 6.**
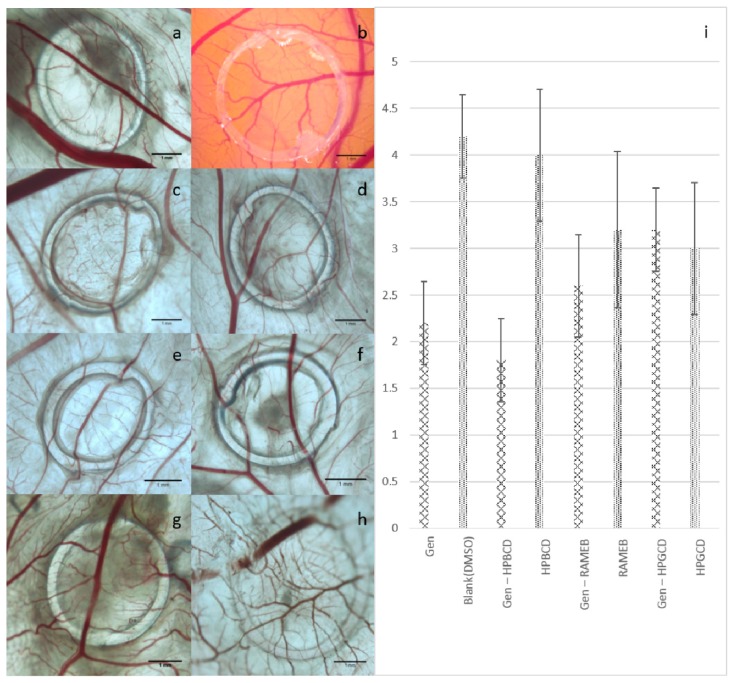
Effects on angiogenesis for genistein (Gen) and the tested complexes prepared with RAMEB (Gen-RAMEB), HPBCD (Gen-HPBCD) and HPGCD (Gen-HPGCD) on the CAM: (**a**–**h**) Stereomicroscopic photographs of *ex ovo* samples, day 5: (**a**) Gen; (**b**) Blank-DMSO; (**c**) Gen-HPBCD; (**d**) HPBCD; (**e**) Gen-RAMEB; (**f**) RAMEB; (**g**) Gen-HPGCD; (**h**) HPGCD; (**i**) Vascular density mean scores induced by the samples on the CAM using 0–5 scale.

All treated specimens showed good viability rates, with longer survival times for Gen complexes, in the following order: HPGCD < HPBCD < RAMEB. On the fifth day (final day) of the experiment (12th day of incubation), the samples were photographed and a morphometric analysis was applied. The samples containing Gen determined a reduction of the vascular density compared to the blank specimen. The photographs of the *ex ovo* samples in the final day of the experiment show the arrangement of the blood vessels around the application area. The samples with higher implications in the angiogenic process present a lower number of capillaries converging toward the ring and significant interruptions in the branching pattern (Figure 6).

Intense antiangiogenic effects were noticed for Gen alone. Even more pronounced was the effect induced by Gen in complex with HPBCD. Important, though lower, effects were determined by the other two Gen complexes. The reduction of vessel density by Gen samples, as evaluated by applying the 0–5 score method and exhibited in Figure 6i, is presented in this order: Gen-HPGCD < Gen-RAMEB < Gen < Gen-HPBCD. The control specimens, represented by CDs alone were also investigated, revealing they only induced a slight decrease in the vascularization, not very different in comparison to the blank samples. The CD that induced the most important reduction of vessel branching was HPGCD, while HPBCD had the lowest inhibitory effect. Statistically significant differences (*p* < 0.05) have been found for the control and the compound group, indicating the presence of biological activity for Gen. The analysis for each Gen formulation and the paired control sample showed statistically significant differences for Gen in DMSO and for Gen in HPBCD, but not for the two other CDs formulations, *i.e.*, Gen-RAMEB *versus* RAMEB (*p* = 0.217) and Gen-HPGCD *versus* HPGCD (*p* = 0.608).

Recently Gen has been found as an antiangiogenic compound both *in vitro* and *in vivo* and on CAM model [52,53]. It is known that isoflavonoids can act through the indirect reduction of VEGF levels or by stimulating the inhibitory pathways of angiogenesis [53]. The precise underlying mechanism is not yet fully understood. Kiriakidis *et al.* [52] tested Gen and daidzein from a red clover extract, in concentrations of 50 μM, and showed on a CAM model a reduction of the vascularized area of around 81% for Gen and only 55% for daidzein [52].

Correlating our results concerning the angiogenesis influence of Gen samples, with the data regarding the stability of the complexes and the inhibitory effects on tumor cells, the complex having an intermediate stability, *i.e*., Gen-HPBCD, seemed to present the strongest activity as angiogenic inhibitor, with optimal tolerability. We have previously investigated HPGCD in complex with betulinic acid, revealing a poor influence of the CD on the antiangiogenic effect of the active compound, being though considered a good option as solubility enhancer [54]. The two complexes, Gen-RAMEB and Gen-HPGCD, showed lower effects compared to pure Gen. Statistical analysis indicates that, compared to the other formulations, Gen in HPBCD highly reduces the vascular density, probable through an appropriate release of the active compound from the complex. Among the investigated CDs the less expensive RAMEB could be further investigated.

## 3. Experimental Section

### 3.1. Reagents

Genistein was purchased from Extrasynthese (Genay, Cedex, France, purity > 95%), hydroxylpropyl-β-cyclodextrin (HPBCD), hydroxypropyl-gamma-cyclodextrin (HPGCD) and randomly-metylated-β-cyclodextrin (RAMEB) from Cyclolab, Budapest, Hungary. All substances were used as received.

### 3.2. Computational Details

The calculations have been performed in the framework of Density Functional Theory (DFT) method. The molecular geometry optimizations and energetic of these complexes were performed with the Gaussian 09 software package [55] by using B97D functional in conjunction with 6-31G(d) basis set [56]. Geometry optimization of these complexes are conducted in gas phase (vacuum), Dimethyl sulfoxide solution (DMSO, ɛ = 46.826) and water (ɛ = 78.355) using the polarizable continuum model (PCM) [57–59].

The interaction energies are computed for all of these three systems in order to characterize the relative energies and to have an overview about the strength of interactions between the guest molecule (Gen) and the three types of hosts systems (HPBCD, HPGCD, RAMEB). These calculations are conducted in gas phase and in DMSO solution.

The interaction energy was calculated as: *E*_interation_ = *E*_AB_(*r*_AB_) − (*E*_A_(*r*_AB_) + *E*_B_(*r*_AB_)) with the energy of the monomers computed using their geometry in the dimer. The basis set superposition error (BSSE) was also determinate using the Counterpoise method (CP). For all the three complexes, the binding mechanism is much influenced by the dispersion interactions between the host and the guest molecules.

### 3.3. Complexes Preparation

Inclusion complexes were prepared by kneading the physical mixture of Gen and cyclodextrin with a 50% (*w*/*w*) water: ethanol solution until the bulk of solvent evaporated and a paste-type product was formed; the mixture was then dried at room temperature for 24 h and put in the oven, at 105 °C, for several hours until reaching constant weight. The final product was pulverized and sieved.

All the binary complexes were prepared using 1:1 genistein:CD molar ratio (*M*w_Gen_ = 270.25, *M*w_HPBCD_ = 1396, *M*w_RAMEB_ = 1303).

### 3.4. Phase Solubility Studies

The water solubility of genistein in the presence of RAMEB, HPBCD and HPGCD, respectively, was studied according to the Higuchi and Connors method [60]. Excess amounts of genistein were added to a range of water solutions containing various concentrations (25–300 mM) of cyclodextrin (RAMEB, HPBCD or HPGCD, respectively). The suspensions were shaken at room temperature for a period of five days, until equilibrium was reached, and then filtered. The clear solutions were then diluted and analyzed by UV spectrophotometry at 270 nm, using the same concentrations of cyclodextrin in water as blanks, in order to compensate the cyclodextrin absorption. The apparent stability constant was calculated for each cyclodextrin according to the following equation:

$$\text{K}=\frac{\text{slope}}{{\text{S}}_{0}\;(1-\text{slope})}$$

where *S*_0_ = Gen water solubility and the slope is the slope of the solubility diagram. All measurements were conducted in triplicate.

### 3.5. Differential Scanning Calorimetry (DSC)

The DSC measurements were made with a Mettler Toledo DSC 821^e^ (Mettler Inc., Schwerzenbach, Switzerland) thermal analysis system with the STAR^e^ thermal analysis program V9.1. Approximately 2–5 mg of genistein or its product was examined in the temperature range between 25 and 350 °C. The heating rate was 5 °C min^−1^. Argon was used as carrier gas, at a flow rate of 10 L h^−1^ during the DSC investigation.

### 3.6. X-ray Diffraction

X-ray-diffraction patterns were obtained on a Philips PW 1710 diffractometer (PW 1930 generator, PW 1820 goniometer, Angstrom Advanced Inc., Braintree, MA, USA), where the tube anode was Cu with *K*α = 1.54242 Å. The pattern was collected with a tube voltage of 50 kV and 40 mA of tube current in step scan mode (step size 0.035, counting time 1 s per step).

### 3.7. Scanning Electron Microscopy (SEM) Assay

The shape and surface characteristics of genistein and complex were visualized using a scanning electron microscope (Hitachi S4700, Hitachi Scientific Ltd., Hitachi, Japan). The samples were sputter coated with gold-palladium under an argon atmosphere using a gold sputter module in a high vacuum evaporator and the samples were examined using SEM set at 15 kV.

### 3.8. MTT *in Vitro* Analysis

Human adherent cell lines were purchased from ECACC (European Collection of Cell Cultures, Salisbury, UK) and were grown in minimal essential medium (MEM) supplemented with 10% heat-inactivated fetal calf serum (FCS), 1% non-essential amino acids and 1% penicillin-streptomycin in a humidified atmosphere containing 5% CO_2_ 37 °C. All cell culture media and supplements were obtained from PAA Laboratories GmbH (Pasching, Austria). Four types of cancer cells lines: HeLa (cervical adenocarcinoma), MCF-7 (breast adenocarcinoma), A2780 (human ovarian carcinoma), A431 (skin epidermoid carcinoma) were seeded onto a 96-well microplate and attached to the bottom of the well during overnight. After 24 h 200 μL of new medium containing the test substances were added and incubated for 72 h. Gen, Gen:RAMEB, Gen:HBPCD and Gen:HPGCD were added in the concentrations of 1, 3, 10, 30, 60 and 90 μM. All three cyclodextrins were also tested alone, in the same range of concentrations. After treatment, the living cells were then assayed by the addition of 20 μL of 5 mg/mL MTT (3-(4,5-dimethylthiazol-2-yl)-2,5-diphenyltetrazolium bromide) solution. The intact mitochondrial reductase converted and precipitated MTT (3-(4,5-dimethylthiazol-2-yl)-2,5- diphenyltetrazolium bromide) as blue crystals during a 4 h contact period. The medium was then removed, and the precipitated crystals were dissolved in 100 μL of dimethyl sulfoxide (DMSO). Finally, the reduced MTT was spectrophotometrically analyzed at 545 nm, using a microplate reader; wells with untreated cells were used as controls [61]. All *in vitro* experiments were carried out on two microplates with at least five parallel wells. DMSO was used to prepare stock solutions of the tested substances and the highest DMSO concentration (0.3%) of the medium did not have any significant effect on the cell proliferation. Sigmoidal dose-response curves were fitted to the measured points, and the *IC*_50_ values were calculated by means of GraphPad Prism 4 (GraphPad Software, San Diego, CA, USA).

### 3.9. *In Vitro* Antibacterial Activity

Gen, Gen:RAMEB, Gen:HBPCD, Gen:HPGCD were screened for their antimicrobial activity against 7 bacterial strains: *Bacillus subtilis* (ATCC 6633), *Enterococcus faecalis* (ATCC 29212), *Escherichia coli* (ATCC 25922), *Salmonella typhimurium* (ATCC 14028), *Shigella sonnei* (ATCC 25931), *Pseudomonas aeruginosa* (ATCC 27853), *Staphylococcus aureus (*ATCC 25923) by the agar disk-diffusion method and the dilution method with MIC (minimal inhibitory concentration) determination.

### 3.10. Disk Diffusion Method

The antimicrobial activity of the above mentioned compounds was evaluated according to the guidelines of the National Committee for Clinical Laboratory Standards (NCCLS, 1997) using agar disk diffusion method. NCCLS recommends a bacterial suspension with a density equal to a 0.5 McFarland (which gives a final bacterial concentration of 1–2 × 10^8^ CFU/mL). The Mueller-Hinton agar plates (Thermo Scientific/Oxoid, Wesel, Germany) were inoculated with bacterial suspension using a sterile cotton swab. Stock solutions of tested compounds were prepared in DMSO at a concentration of 10 mM. Within 15 min after the plates were inoculated, sterile Whatman No. 1 filter paper disks (6 mm in diameter) (Sigma Aldrich, Taufkirchen bei Munchen, Germany) impregnated with 20 μL solution of tested compounds were distributed evenly on the surface, with at least 30 mm (center to center) between them. CDs alone were also tested as control for the same range of concentrations. Plates inoculated with the bacterial suspensions were incubated at 37 °C for 24 h. The inhibition zone diameters were measured in millimeters, with a ruler. For all bacterial strains, we performed triplicate disk-diffusion tests and the results were expressed as the mean-value ± SD.

### 3.11. Dilution Assay for Minimal Inhibitory Concentration (MIC) Determinations

All the bacterial strains used in this experiment were prepared from 24 h cultures. Suspensions were adjusted to a McFarland’s standard of 0.5 (which gives a final bacterial concentration approximately 10^8^ CFU/mL) and diluted 1:200 in nutrient medium, then incubated at 37 °C. This resulted in approximately 500,000 CFU/mL. In glass test tubes, a 200 μL bacterial suspension was distributed to 200 μL of test medium containing serial two-fold dilutions of test compounds. These tubes were incubated for 18 h at 37 °C. Gen, Gen:RAMEB, Gen:HBPCD, Gen:HPGCD evaluated concentrations were 0.63, 1.25, 2.5, 5 and 10 mM. CD’s alone were also tested as control for the same range of concentrations. The MIC (Minimal Inhibitory Concentrations) were recorded as the minimum concentration of the compound which inhibited the visible growth of the tested microorganism. For all bacterial strains, we performed the experiment three times.

### 3.12. The Chorioallantoic Membrane Assay (CAM Assay)

The method uses as biologic material fertilized chicken eggs (*Gallus gallus domesticus*). They are prepared for the experiment by cleaning with ethanol dating and then placing them horizontally in an incubator at 37 °C, with constant humidity. On the third day of incubation, 3–4 mL of albumen were extracted from the most pointed part of the egg, so that the chorioallantoic developing membrane can detach from the inner shell in order to be able to work directly on the membrane. The following day (day 4 of incubation) a window is cut on the surface of the egg, resealed with adhesive tape and incubation goes on until the day of the experiment [62,63].

The genistein samples were tested for their implications in the process of angiogenesis during the period of rapid growth of the blood vessels (day 7–day 12 of incubation). The evaluation was performed *in ovo*, from the seventh day of incubation, by pipetting 3 doses of 3 μL (on day 7, day 9 and day 11 of incubation) from each test and control solutions on top of the chorioallantoic membrane inside a sterile plastic ring. The samples were represented by Gen, Blank-DMSO (1%), Gen:HPBCD (1:1), Gen:HPGCD (1:1), Gen:RAMEB (1:1), HPBCD, HPGCD, RAMEB. The concentration for each sample was 10 mM. All samples were applied in triplicate. The treated CAMs were daily investigated *in ovo* by means of stereomicroscope (Zeiss Axio V16 Stereomicroscope, Carl Zeiss, Jena, Germany and all images were recorded with Axio CAM and Zeiss ZEN software (Carl Zeiss, Jena, Germany). On the final day of the experiment, *i.e.*, day 12 of incubation, the tested membranes were collected in buffered formalin and photographed in a petri dish. These images were used for the morphometric evaluation. The method applied consisted in scoring the intensity of the vascular density towards the ring using an arbitrary 0–5 scale [64]. A high score indicated normal ongoing angiogenesis, while the lower values were related to a deregulated process. The results were expressed as mean values ± standard deviation. All data was statistical analyzed, using SPSS software (IBM SPSS Statistics for Windows, Version 20.0.; IBM Corp., Armonk, NY, USA). The one-way ANNOVA test was carried out for the comparison between the two big groups, *i.e*., control group (DMSO, the three cyclodextrins) and compound group (Gen alone and Gen incorporated in the three CDs, respectively). The same statistical test was applied in order to compare each type of compound formulation with the pair control sample. A *p*-value of ≤0.05 was considered to be of statistical significance.

## 4. Conclusions

Having the complete picture of the tested biological activities for Gen alone and Gen incorporated in the three CDs, respectively, we can adjudge that a direct and tight correlation between theory and all biological activity for all analyzed *in vitro* and *in vivo* aspects does not exist. This might be due to the complexity and specificity of each individual tested biological aspect, the antiproliferative, antimicrobial and antiangiogenic mechanisms and particularities. Computational results suggest that the complex of genistein with randomly methylated β-cyclodextrin (RAMEB) Gen:RAMEB would be the most proper complex in terms of both Gen water solubility and lower interaction energy. “Real” and in a way assumable, the analyzed samples act differently depending on the tested system, as explained in the paper. However, we can state that complexation of Gen in a molar ratio of 1:1 with the mentioned ramified cyclodextrins is a good method to modulate water solubility and improve the biological activity.

## Figures and Tables

**Table 1.** Interaction energy of the three complexes.

Solvent	Gen-RAMEB		Gen-HPBCD		Gen-HPGCD	
		
	*E*_int_ (Non-CP a) (kcal/mol)	*E*_int_ (CP b) (kcal/mol)	*E*_int_ (Non-CP a) (kcal/mol)	*E*_int_ (CP b) (kcal/mol)	*E*_int_ (Non-CP a) (kcal/mol)	*E*_int_ (CP b) (kcal/mol)
Gas Phase	−34.68	−21.32	−50.14	−33.15	−54.25	−37.08

DMSO	−30.07	−19.55	−43.84	−31.01	−48.38	−36.27

Water	−29.83	−20.29	−43.79	−30.98	−48.02	−36.18

a , Non-counterpoise correction;

b , Counterpoise correction.

**Table 2.** Stability constants for Gen complexes.

Cyclodextrin	Stability constant (M^−1^)
RAMEB	10.850
HPBCD	10.900
HPGCD	12.700

**Table 3.** Calculated antiproliferative *IC*_50_ values for genistein and the tested complexes on four different human cancer cell lines.

Compound	*IC*_50_ values (μM) a			

	HeLa	A2780	MCF7	A431
Gen	25.90	20.86	50.08	72.79
Gen-RAMEB (1:1)	21.07	22.80	59.47	>90
Gen-HPBCD (1:1)	16.47	17.49	44.02	69.88
Gen-HPGCD (1:1)	19.09	19.84	50.40	74.35

a *IC*_50_ represent the concentration of active agent necessarily to reduce proliferation to 50%. Mean value from two independent determinations with five parallel wells, standard deviation less than 15%.

**Table 4.** Zone of inhibition (mm) for genistein and the tested complexes on the mentioned bacterial strains.

Compound	Zone of inhibition (mm). Results are presented as mean values ± SD						

	*B. subtilis*	*S. aureus*	*E. faecalis*	*E. coli*	*S. typhimurium*	*S. sonnei*	*P. aeruginosa*
Gen	15 ± 0.46	9 ± 0.12	7 ± 0.23	6 ± 0.23	6 ± 0.15	6 ± 0.23	6 ± 0.12
Gen-RAMEB (1:1)	16 ± 0.32	9 ± 0.17	8 ± 0.19	6 ± 0.14	6 ± 0.13	6 ± 0.09	6 ± 0.17
Gen-HPBCD (1:1)	16 ± 0.20	9 ± 0.11	8 ± 0.13	6 ± 0.21	6 ± 0.19	6 ± 0.14	6 ± 0.22
Gen-HPGCD (1:1)	17 ± 0.28	9 ± 0.09	8 ± 0.11	6 ± 0.16	6 ± 0.17	6 ± 0.15	6 ± 0.13
